# Mild to Moderate Sleep Restriction Does Not Affect the Cortisol Awakening Response in Healthy Adult Males

**DOI:** 10.3390/clockssleep4040054

**Published:** 2022-11-25

**Authors:** Thomas G. Kontou, Gregory D. Roach, Charli Sargent

**Affiliations:** The Appleton Institute for Behavioural Science, CQ University, Wayville, SA 5034, Australia

**Keywords:** salivary cortisol, sleep duration, time in bed, cortisol area under the curve, healthy males, sleep restriction, anticipation

## Abstract

The cortisol awakening response (CAR) is a distinct rise in cortisol that occurs upon awakening that is thought to contribute to arousal, energy boosting, and anticipation. There is some evidence to suggest that inadequate sleep may alter the CAR, but the relationship between sleep duration and CAR has not been systematically examined. Healthy males (*n* = 111; age: 23.0 ± 3.6 yrs) spent 10 consecutive days/nights in a sleep laboratory. After a baseline night (9 h time in bed), participants spent either 5 h (*n* = 19), 6 h (*n* = 23), 7 h (*n* = 16), 8 h (*n* = 27), or 9 h (*n* = 26) in bed for seven nights, followed by a 9 h recovery sleep. The saliva samples for cortisol assay were collected at 08:00 h, 08:30 h and 08:45 h at baseline, on experimental days 2 and 5 and on the recovery day. The primary dependent variables were the cortisol concentration at awakening (08:00 h) and the cortisol area under the curve (AUC). There was no effect of time in bed on either the cortisol concentration at awakening or cortisol AUC. In all the time in bed conditions, the cortisol AUC tended to be higher at baseline and lower on experimental day 5. Five consecutive nights of mild to moderate sleep restriction does not appear to affect the CAR in healthy male adults.

## 1. Introduction

The cortisol awakening response (CAR) is a distinct rise in cortisol that occurs upon awakening [1]. It is characterised by a from 50 to 160% increase in cortisol in the first 30 to 45 min after a sleep period [2,3,4,5]. The magnitude of the CAR can vary based on situational factors. For example, the CAR is higher in individuals who are anticipating a stressful day [6] or experiencing stress at work [7]. Conversely, a lower CAR may be indicative of HPA axis dysfunction [8] and is often observed in individuals living with chronic conditions including post-traumatic stress disorder, chronic stress [9], or type 2 diabetes [10,11]. The CAR only occurs upon awakening (particularly after a night-time sleep period) and, thus, its link with sleep is inextricable [12,13]. Altering sleep duration may have an important influence on CAR, however, the nature of this influence has not been systematically examined under experimental conditions.

Under some circumstances, behavioural sleep restriction (i.e., sleep that is restricted due to lifestyle factors such as going to bed late and/or waking up early), has been associated with a high CAR. In field-based studies, in otherwise healthy individuals, shorter subjective total sleep time (i.e., five hours of time in bed) is associated with a higher CAR area under the curve compared to sleeping more than five hours per night [14,15]. This higher area under the curve occurs despite the initial cortisol sample (extracted upon awakening) being lower in short sleepers than it is in long sleepers. These findings indicate an association between sleep duration and the cortisol awakening response. However, the saliva samples were collected by participants at home, without supervision from a researcher. When participants self-sample the saliva, delays in sampling often occur [16,17], which can influence the profile of the CAR area under the curve [3,18]. Furthermore, in field studies, it is unclear what other unique factors may influence the CAR in people who identify as short sleepers. For example, the possible reasons for short sleep may also be a source of stress, such as a high workload [19,20].

Sleep restriction that is induced experimentally has an influence on daily cortisol concentrations. Specifically, when sleep is restricted to five hours per night and cortisol is measured regularly, cortisol concentrations are lower in the morning [21,22,23,24] and higher in the afternoon [25,26,27] than after a normal night of sleep (i.e., from eight to ten hours of time in bed). However, when morning cortisol concentrations were lower following sleep restriction, the participants were woken at least two hours earlier in the sleep restriction condition than in the comparison conditions. This approach may confound the effect of time of day (i.e., the circadian system) on morning cortisol with sleep restriction—such that for normally entrained individuals, at an earlier time in the morning, the cortisol concentrations are naturally lower than they are at a later time in the morning [13].

When the time of waking in the morning is identical following a night of sleep restriction and a normal night of sleep, the impact of sleep restriction on the CAR is less clear. For example, the cortisol concentration in the morning is either not different between conditions [28,29,30], lower after sleep restriction [21], or there is a delay in peak cortisol after sleep restriction [31]. The discrepancy in results could be explained by differences in the timing of sampling to measure cortisol. In some protocols, the cortisol was not measured at awakening and only measured every 30 min–1 h after awakening [21,22,28]. In other protocols, the cortisol was measured at awakening and only every 30 min thereafter (e.g., [29]). To ensure the CAR peak is captured, the sampling for CAR must occur at least three times in the first 45 min after awakening (e.g., time 0, time +30, time +45 min) [32]. The approaches where cortisol is measured at awakening and then every 30 min thereafter may not capture the cortisol peak and, therefore, not measure CAR.

The relationship between the sleep restriction and CAR has not been systematically evaluated in an experimental setting. The field studies investigating this relationship have shown mixed results that may be influenced by external factors. The experimental investigations have been designed with the aim of measuring the influence of sleep restriction on overall cortisol concentrations but not for the CAR. As a potential indicator of HPA axis function [6], it is important to understand the influence of sleep restriction on the CAR. Thus, the aim of the present study was to investigate the influence of sleep restriction on the CAR in healthy young males. Five different time in bed conditions were examined (i.e., 9, 8, 7, 6, and 5 h time in bed) and cortisol samples were frequently collected after awakening; at the same three time points in each condition (i.e., 08:00 h, 08:30 h, and 08:45 h). Under these conditions, it is hypothesised that CAR in healthy young males will be lower following 5–7 h time in bed than following 8 or 9 h time in bed.

## 2. Results

### 2.1. Sleep

The participants obtained less sleep when the time in bed was restricted. This reduction in total sleep time was proportional to time spent in bed (Table 1).

### 2.2. Cortisol at Awakening (08:00 h)

Most of the data sets for cortisol at awakening were not normally distributed. A log transformation addressed this issue, except for BL in the 7 h condition (*W*_16_ = 0.88, *p* = 0.044) and E2 in the 5 h condition (*W*_21_ = 0.84, *p* = 0.003). The assumption of sphericity was violated (χ^2^_5_ = 14.5, *p* = 0.013) and a Greenhouse Geiser correction was applied. The homogeneity of variance was violated on BL (*F*_4, 106_ = 2.52, *p* = 0.045). The statistical outliers were present but were not removed from the analyses because they were not outside the physiological range for cortisol (0.3–70.1 nmol·L^−1^) [33].

**Figure 1:**
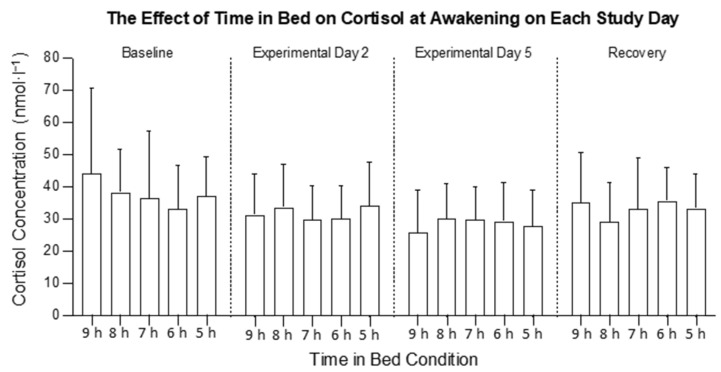
The effect of time in bed on mean cortisol concentration at awakening (08:00 h) in the 9 h, 8 h, 7 h, 6 h, and 5 h conditions on each study day. The columns are means and the error bars are standard deviations.

**Figure 2:**
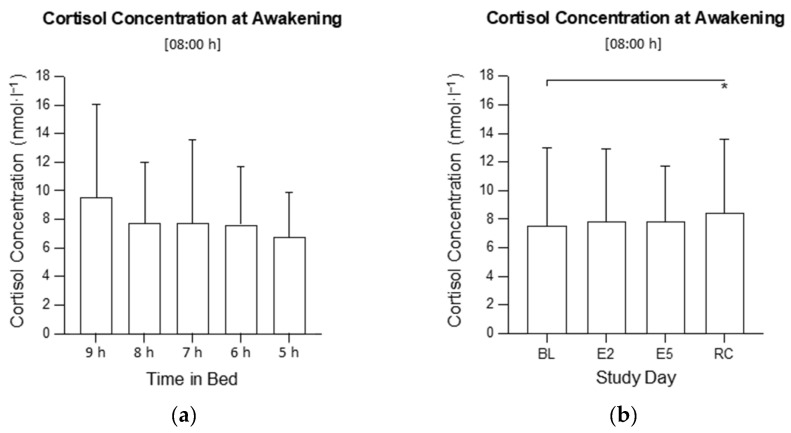
(**a**) Main effect of time in bed on mean cortisol concentration at awakening (08:00 h) in the 9 h, 8 h, 7 h, 6 h, and 5 h conditions; (**b**) Main effect of study day on mean cortisol concentration at awakening on baseline day 1 (BL), experimental day 2 (E2), experimental day 5 (E5), and the recovery day (RC). * indicates *p* < 0.05. The columns are means and the error bars are standard deviations.

There was no interaction between the time in bed and study day on the cortisol concentration at 08:00 h (*F*_12, 318_ = 1.20, *p* = 0.287; Figure 1) and no main effect of time in bed on the cortisol concentration at 08:00 h (*F*_4, 106_ = 1.18; *p* = 0.324; Figure 2a), but there was a main effect of the study day (*F*_3, 318_ = 4.02, *p* = 0.008; Figure 2b). The cortisol concentration at 08:00 h was higher on the RC compared with the BL (*p* = 0.022).

### 2.3. Cortisol Area under the Curve (AUC)

Most of the data sets for cortisol area AUC were not normally distributed. A log transformation addressed this issue, except for E2 in the 7 h condition (*W*_16_ = 0.88, *p* = 0.041) and the RC in the 6 h condition (*W*_23_ = 0.91, *p* = 0.032). The statistical outliers were present but were not removed because the raw values were not outside the physiological range for cortisol (0.3–70.1 nmol·L^−1^) [33]. The assumption of sphericity was violated (χ^2^_5_ = 18.9, *p* = 0.002) and a Greenhouse Geiser correction was applied. The assumption of homogeneity of variance was met on all study days.

**Figure 3:**
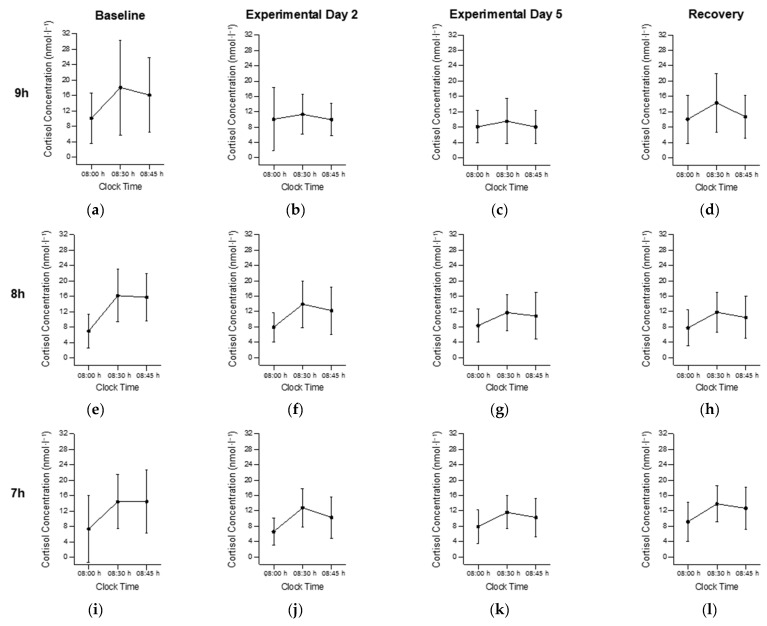

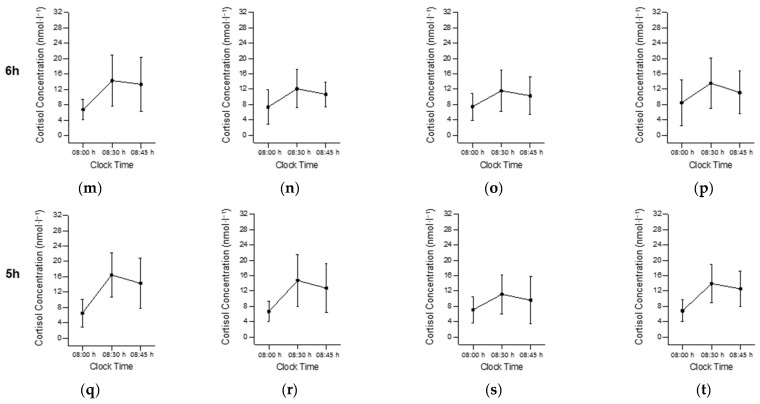
The cortisol awakening response across study days in the 9 h condition (**a**–**d**), the 8 h condition (**e**–**h**), the 7 h condition (**i**–**l**), the 6 h condition (**m**–**p**), and the 5 h condition (**q**–**t**). Data mean ± standard deviation.

**Figure 4:**
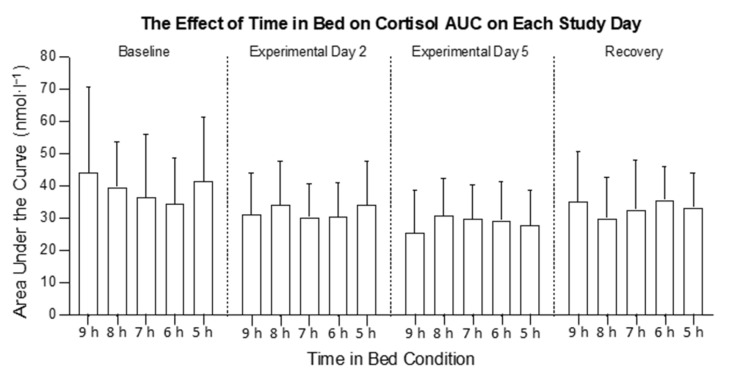
The effect of time in bed on cortisol area under the curve (AUC) in the 9 h, 8 h, 7 h, 6 h, and 5 h conditions on each study day. The columns are means and the error bars are standard deviations.

**Figure 5:**
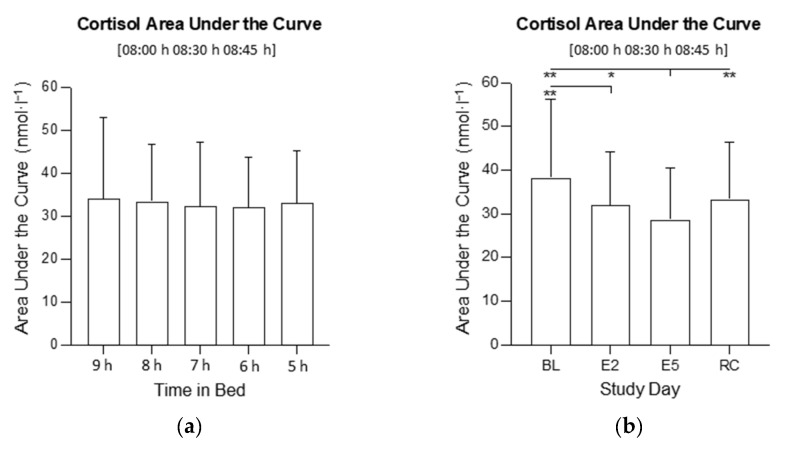
(**a**) Main effect of time in bed on mean cortisol AUC in the 9 h, 8 h, 7 h, 6 h, and 5 h conditions; (**b**) Main effect of study day on mean cortisol AUC on baseline day 1 (BL), experimental day 2 (E2), experimental day 5 (E5), and the recovery day (RC). * indicates *p* < 0.05, ** indicates *p* < 0.01. The columns are means and error bars are standard deviations.

There was an interaction between time in bed and study day for cortisol AUC (*F*_12,318_ = 2.56, *p* = 0.005; Figure 3 and Figure 4). The visual inspection indicated that the cortisol AUC was higher on the BL and lower on E5 in all conditions, but the post hoc comparisons were not significant. There was no main effect of time in bed on cortisol AUC (*F*_4, 106_ = 0.10; *p* = 0.981; Figure 5a), but there was a main effect of the study day (*F*_3, 318_ = 17.1, *p* < 0.001; Figure 5b). On E5, the cortisol AUC was lower compared with the BL (*p* < 0.001), E2 (*p* = 0.013) and the RC (*p* < 0.001). On E2, the cortisol AUC was lower compared with the BL (*p* < 0.001).

### 2.4. Cortisol Mean Increase (MnInc)

Most of the data sets for MnInc cortisol were normally distributed, except for E2 in the 6 h condition (*W*_23_ = 0.91, *p* < 0.039), E2 in the 9 h condition (*W*_24_ = 0.82, *p* < 0.001), and E5 in the 5 h condition (*W*_21_ = 0.86, *p* < 0.007). The statistical outliers were present but were not removed because the raw values were not outside the physiological range for cortisol (0.3–70.1 nmol·L^−1^) [33]. The assumption of sphericity was violated (χ^2^_5_ = 14.5, *p* = 0.013) and a Greenhouse Geiser correction was applied. The assumption of homogeneity of variance was only violated on the BL (F_4, 106_ = 2.52, *p =* 0.045).

**Figure 6:**
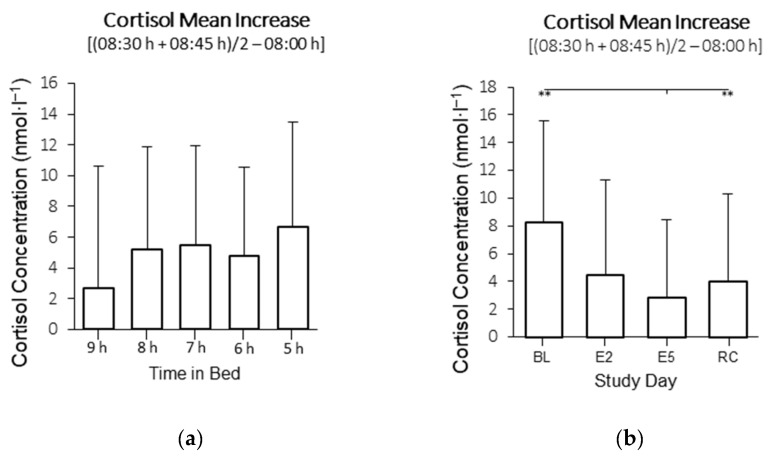
(**a**) Main effect of time in bed on cortisol mean increase (08:30 h + 08:45 h)/2 − 08:00 h) in the 9 h, 8 h, 7 h, 6 h, and 5 h conditions; (**b**) Main effect of study day on cortisol mean increase at awakening on baseline day 1 (BL), experimental day 2 (E2), experimental day 5 (E5), and the recovery day (RC). ** indicates *p* < 0.01. The columns are means and the error bars are standard deviations.

There was no interaction between time in bed and study day for cortisol MnInc (*F*_10.36, 318_ = 0.90 *p* = 0.535). There was no main effect of time in bed on cortisol MnInc (*F*_4, 106_ = 2.01; *p* = 0.098; Figure 6a), but there was a main effect of the study day (*F*_2.591, 106_ = 17.87, *p* < 0.001; Figure 6b). On E5, the cortisol MnInc was lower compared with the BL (*p* < 0.001), and RC (*p* < 0.001).

## 3. Discussion

The aim of the present study was to investigate the impact of moderate to mild sleep restriction on the cortisol awakening response (CAR). The main finding of the study was that spending 5–7 h time in bed for five consecutive nights did not affect the CAR when assessed over the first 45 min after waking. The lack of influence of sleep restriction on the CAR in the present study is in contrast with findings reported in field-based studies examining short sleepers. Habitual short sleep (i.e., less than 6 h per night) has been associated with a lower CAR [34,35] or a higher CAR [14,15]. However, in field studies, the effects of sleep restriction on the CAR are not isolated from the potential impact of other sleep or lifestyle-related stressors on the CAR (e.g., obstructive sleep apnoea, insomnia, a high workload, or social/familial demands) and, therefore, causality cannot be confidently inferred [14]. Furthermore, total sleep time in field-based studies is typically measured using self-reporting [15], which raises the possibility of under- or overreporting total sleep time [36]. These variabilities cause these results to be difficult to interpret. In the present study, the time in bed was controlled and the time of waking in the morning was consistent across all time in bed conditions. The results indicate that under such conditions, there is no effect of consecutive nights of moderate to mild sleep restriction on the CAR.

The magnitude of sleep restriction employed in the present study ranged from moderate (i.e., 5 h time in bed) to mild (i.e., from 6 h to 7 h time in bed). It is possible that more severe sleep restriction (i.e., <5 h time in bed) may have altered the CAR. For example, when the time in bed is restricted to 3 h per night compared with 10 h per night, the morning cortisol concentration is reduced [21]. However, when the time in bed is restricted to 4 h per night compared with 8 h per night, the morning cortisol concentration is not different [29]. Although the cortisol concentration was not sampled frequently enough to determine the CAR in the aforementioned studies [21,29], the results suggest that 4 h time in bed may be a threshold below which morning cortisol concentrations become impaired. Importantly, in the present study, it appears that in healthy young males, the CAR is robust in mild sleep restriction. Therefore, in epidemiological studies, when mild sleep restriction has been associated with a lower CAR, the construct leading to an impaired CAR may not be sleep restriction [34]. It is possible that other stressors that lead to sleep restriction (e.g., anticipating a day with high demands) is what may also lead to an elevated CAR. In the future, it will be important to determine whether the CAR is influenced by more severe sleep restriction (i.e., <4 h time in bed) or experimentally induced sleep fragmentation.

In the present study, the CAR (as measured by the area under the curve and mean increase) was highest at the start and end of the protocol. Although the precise function and purpose of the CAR remains unclear, there is some evidence to suggest that the CAR has potential roles within the processes of arousal, energy boosting, and anticipation [37]. For example, the CAR is typically higher when individuals are anticipating a day with high demands (e.g., a workday) compared to a day with low demands (e.g., a rest day) [37,38]. In the context of the present study, a high CAR at the start and end of the protocol may reflect an “anticipatory” response—i.e., the CAR is high on the baseline morning as participants prepare for the experimental protocol and the CAR is high on the recovery morning as participants prepare to exit the laboratory and return home. The reason for the higher CAR on the first morning of the protocol may also be attributable to the first night effect. The first night effect (described as disruption to sleep physiology on the first night of a live-in sleep study) can result in changes to some aspects of the architecture [39] (however, sleep onset latency, total sleep time, and wake after sleep onset are unaffected [40]) and may influence cortisol concentrations [41]. The potential influence of the “first night effect” is a limitation of the study and future studies may benefit from recording baseline CAR after a second baseline night. Such a methodological approach may also reduce the intra-individual variability that was observed in the present study as the magnitude of the first night effect can vary between individuals [39]. Subjective measures of anticipation, stress, and daily demands were not collected in the present study, so it is not possible to determine whether the participants did indeed perceive the demands of the protocol differently on different days. In the future, it may be useful to obtain such measurements when assessing the impact of experimental sleep restriction on the CAR.

The findings of the present study must be considered in the context of the boundary conditions of the protocol. The time in bed was restricted for seven consecutive nights and cortisol was sampled on the morning after baseline, after two and five nights of sleep restriction, and after the recovery sleep. It is possible that changes in the CAR may occur after longer periods of sleep restriction such as multiple weeks, months, or years—as has been reported in longitudinal studies [14,15]. However, such an approach is not usually practical in a continuous laboratory study. In the present study, other than measuring the CAR, no other objective or subjective measures of arousal, anticipation, or demands were assessed. Thus, it was not possible to determine if sleep restriction or other aspects of the laboratory environment affected the perceived daily demand. Indeed, gaining insights into participants’ interpretations of daily demands may have allowed for some interpretation of the large intra-individual variability that occurred. A crucial aspect of the present study was the methodological approach to determine the CAR. The cortisol was measured at three time points after awakening (e.g., time 0, time +30, time +45 min) allowing for an accurate determination of the CAR area under the curve [32]. Additionally, any difference between conditions brought about by the circadian influence on the CAR was minimised by (i) dimming the lights in participant areas from 23:00 h until bedtime in the sleep restriction conditions and (ii) achieving sleep restriction by delaying bedtime, which allowed for consistent waking and saliva sampling times between conditions. It should be noted that bed and wake times were not tailored to participants’ usual routines but were fixed (08:00 h wake up on all days in all conditions). This may have resulted in some circadian advance (or delay) on the first one to two nights of the protocol.

## 4. Materials and Methods

### 4.1. Participants

The healthy male adults (*n* = 111) provided written, informed consent to participate in the study. The participants had a mean (±SD) age of 23.0 (±3.7) years and a mean body mass index of 22.9 (±2.0) kg·m^−^². The participants were non-smokers and did not suffer from any metabolic or psychological disorders. The participants were not on a diet, had not experienced any significant weight loss or gain in the three months prior to study commencement, and did not consume excessive amounts of alcohol or caffeine. The self-report questionnaires were used to determine that participants were not suffering (previously or currently) from any sleep disorders and had not undertaken shift work in the three months prior to the study. The participants wore activity monitors for seven nights immediately prior to study commencement to determine whether they obtained a minimum of 7.0 h per night and were going to bed before midnight. The participants were well rested prior to participation, achieving 8.0 ± 0.8 h (mean ± SD) of time in bed in the seven nights prior to study commencement. The interested participants completed a screening health questionnaire and eligible participants were invited to the sleep laboratory for a familiarization session. The project was approved by the Central Queensland University Ethics Committee (H14/11-249). The data in the present study were collected as part of a larger study and, to satisfy recruitment criteria of the larger study, only male participants were selected.

### 4.2. Experimental Design

**Figure 7:**
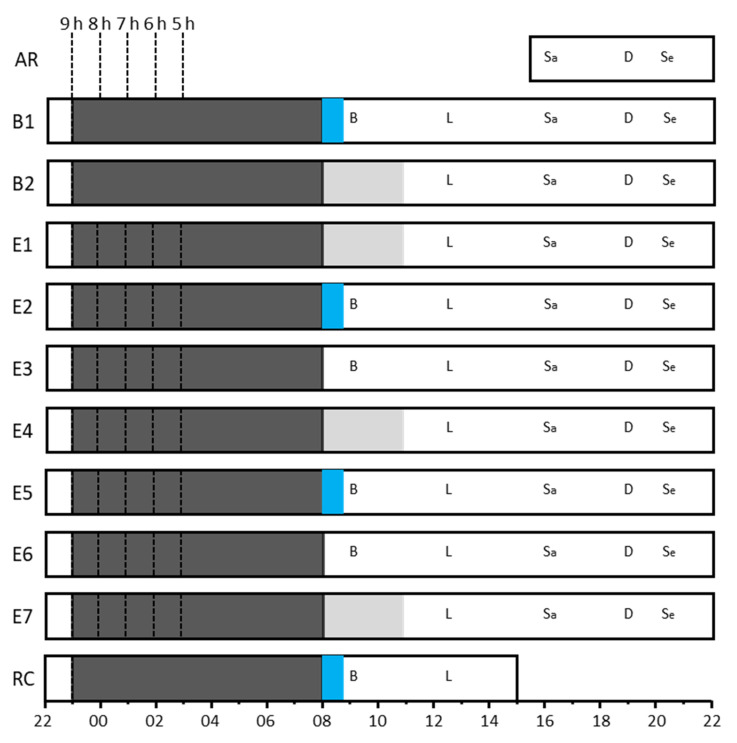
Study protocol. The x-axis indicates clock time (hh) from 22:00 h to 22:00 h. AR = arrival day; B1 and B2 = baseline days 1 and 2; E1 to E7 = experimental days from 1 to 7; RC = recovery day. The blue boxes indicate the duration (from 08:00 h to 08:45 h) over which the saliva samples were collected for cortisol assay. The vertical dashed lines indicate bedtimes for each time in bed condition and the solid dark grey horizontal bars represent time in bed. Solid light grey areas indicate an oral glucose tolerance test (data reported elsewhere).

The study employed a between-groups, repeated measures design in which the participants spent 10 consecutive nights in a sleep laboratory. On the first two nights (B1 and B2, Figure 7), the participants were allocated nine hours of time in bed (23:00 h–08:00 h). For the next seven nights, the participants were randomly assigned to one of five conditions with either 9 h (*n* = 26), 8 h (*n* = 27), 7 h (*n* = 16), 6 h (*n* = 23), or 5 h (*n* = 19) time in bed beginning at 23:00 h, 00:00 h, 01:00 h, 02:00 h, and 03:00 h each night, respectively. The participants were woken at 08:00 h in the morning on all days in all conditions. The protocol ended the day after a single 9 h recovery sleep (23:00 h–08:00 h). On all nights of the study, in all time in bed conditions, at approximately 21:00 h, the participants had electrodes attached to their scalps and faces. These were necessary to capture polysomnographic recordings of their sleep. The saliva samples for measuring cortisol were collected at 08:00 h, 08:30 h, and 08:45 h on baseline day 1 (BL), experimental days 2 (E2) and 5 (E5), and the recovery day (RC). The data presented here are part of a larger study examining the impact of time in bed on glucose tolerance [42,43].

### 4.3. Laboratory Setting

The participants lived in the sleep laboratory located at the Appleton Institute for Behavioural Science. The laboratory contains six studio-style bedrooms with adjacent living areas, six bathrooms, a kitchen, and a communal dining area. The target ambient temperature of the laboratory was 21–23 °C. During the wake periods between 08:00 h and 23:00 h, the light levels remained constant at ~200 lux. In the 5 h, 6 h, 7 h, and 8 h time in bed conditions (i.e., with bedtimes later than 23:00 h), the light levels were dimmed to ~15 lux until bedtime to prevent phase delays in the timing of the circadian system [44]. During the time in bed, the lights were extinguished (i.e., <0.3 lux).

### 4.4. Protocol

The participants arrived at the sleep laboratory at 16:00 h on the arrival day (AR) and remained in the laboratory until 16:00 h on the recovery day (RC) (Figure 6). The saliva samples for measuring cortisol were collected at 08:00 h, 08:30 h, and 08:45 h on days B1, E2, E5, and RC. When the lights were turned on at 08:00 h, the participants were instructed to sit on the edge of their beds (without standing) and were handed a Salivette by a researcher. The participants opened the Salivette and placed the synthetic swab in their mouths. The participants then gently rolled the swab in their mouths for approximately two minutes before replacing the swab in the tube and attaching the lid. At no point throughout the saliva sampling process did researchers or participants directly handle the synthetic swab.

Once the 08:00 h saliva sample was returned to a researcher, the participants were able to drink water and ambulate to their bathrooms. During this visit to the bathroom, the participants were not permitted to brush their teeth or drink any water. The participants returned to their rooms by 08:10 h to sit in their lounge chair and remained seated until after both the 08:30 h and 08:45 h saliva samples were collected by a researcher. This ensured a continuous, seated posture for at least 20 min before collecting saliva samples to avoid changes in cortisol concentrations attributable to postural change [32]. The participants were trained on the saliva sampling collection procedures at 18:30 h on the arrival day. The saliva samples were refrigerated immediately after collection. The samples were spun in a refrigerated (4 °C) centrifuge (Universal 320R, Hettich, Tuttlingen, Germany) at 1000× *g* for two minutes within two hours of collection. The saliva samples were stored at 20 °C until they were assayed for cortisol.

### 4.5. Measures

The cortisol was assayed from the saliva samples collected with Salivettes (Sarstedt AG & Co, Nümbrecht, Germany). Each Salivette consisted of an outer tube with a lid and contained a synthetic swab. The Salivettes were designed to collect a minimum of 1 mL of saliva per sample. The dependent variables related to cortisol included the cortisol concentration at awakening (08:00 h) and the cortisol AUC (the sum of three consecutive cortisol measurements at 08:00 h, 08:30 h, and 08:45 h).

The total sleep time was assessed using polysomnography (PSG; Compumedics Grael; Abbotsford, Australia). The electrode montage included two electroencephalograms, two electrooculograms, and two electromyograms. The sleep records were analysed by a single technician in 30 s epochs, using established criteria [45].

### 4.6. Analyses

The separate mixed-effects ANOVAs were conducted to examine the impact of time in bed on the awakening cortisol concentration (08:00 h), the cortisol AUC, and the cortisol MnInc ((08:30 h + 08:45 h)/2 − 08:00 h). The model included “time in bed” as a between-groups factor (five levels: 9 h, 8 h, 7 h, 6 h, and 5 h time in bed) and “study day” as a within groups factor (four levels: BL, E2, E5, and RC). All the assumptions of the mixed ANOVA were assessed. All the levels of the independent variables (time in bed and study day) in both dependent variable data sets were tested for normality using the Shapiro–Wilk test. The datasets that were not normally distributed were log transformed prior to analysis, but original values are presented in the figures. The statistical outliers were detected by identifying data points that were further than two standard deviations from the mean; violations of sphericity were assessed using Mauchly’s *W*; and homogeneity of variance was assessed using Levene’s Test for Equality of Variances. The post hoc tests with a Bonferroni correction were applied to identify the direction of any significant main and interaction effects. All the analyses were conducted using SPSS version 27 (IBM Corp, Armonk, NY, USA) with a significance level of *p* = 0.05.

## 5. Conclusions

In the present study, five consecutive nights of mild to moderate sleep restriction did not influence the cortisol awakening response. This finding was obtained in a group of healthy young adult males under controlled laboratory conditions. The cortisol awakening response was higher at the start and end of the protocol, indicating a possible influence of anticipatory demands in the preparation of the protocol and in the preparation for exiting the laboratory. Future research may focus on the effects of more severe sleep restriction (i.e., <5 h) on the cortisol awakening response in healthy young participants and/or after more than seven nights of sleep restriction. This approach, coupled with additional physiological and psychological measures of arousal, anticipation, or daily demands, would allow for important insights into the influence of sleep restriction on the CAR.

## Figures and Tables

**Table 1:** Table indicates the total sleep time (M ± SD) in minutes on each of the study nights in each time in bed condition.

Condition	BL	E2	E5	RC
9 h	472.7 ± 53.5	481.0 ± 37.2	458.2 ± 48.8	462.5 ± 31.5
8 h	483.6 ± 39.8	449.7 ± 9.9	444.0 ± 23.2	469.4 ± 39.5
7 h	473.5 ± 45.1	400.8 ± 21.0	401.6 ± 20.5	479.7 ± 44.6
6 h	462.8 ± 46.3	342.8 ± 13.3	345.1 ± 10.0	484.6 ± 59.6
5 h	487.4 ± 42.7	288.7 ± 4.4	291.0 ± 6.1	512.1 ± 20.0

BL = baseline, E2 = experimental night 2, E5 = experimental night 5, RC = recovery night.

## Data Availability

The datasets generated from the study are available from the corresponding author on reasonable request.
